# Comparison of Clinical Characteristics and Predictors of Mortality between Direct and Indirect ARDS

**DOI:** 10.3390/medicina58111563

**Published:** 2022-10-30

**Authors:** Wen Tang, Rui Tang, Yan Zhao, Junnan Peng, Daoxin Wang

**Affiliations:** Department of Respiratory Medicine, The Second Affiliated Hospital of Chongqing Medical University, Chongqing 400010, China

**Keywords:** ARDS, direct, indirect, 28-day mortality, predictor

## Abstract

*Background and Objectives*: Acute Respiratory Distress Syndrome (ARDS) is a heterogeneous syndrome that encompasses lung injury from a direct pulmonary or indirect systemic insult. Studies have shown that direct and indirect ARDS differ in their pathophysiologic process. In this study, we aimed to compare the different clinical characteristics and predictors of 28-day mortality between direct and indirect ARDS. *Materials and Methods*: The data of 1291 ARDS patients from September 2012 to December 2021 at the Second Affiliated Hospital of Chongqing Medical University were reviewed. We enrolled 451 ARDS patients in our study through inclusion and exclusion criteria. According to the risk factors, each patient was divided into direct (*n* = 239) or indirect (*n* = 212) ARDS groups. The primary outcome was 28-day mortality. *Results*: The patients with direct ARDS were more likely to be older (*p* < 0.001) and male (*p* = 0.009) and have more comorbidity (*p* < 0.05) and higher 28-day mortality (*p* < 0.001) than those with indirect ARDS. Age and multiple organ dysfunction syndrome (MODS) were predictors of 28-day mortality in the direct ARDS group, while age, MODS, creatinine, prothrombin time (PT), and oxygenation index (OI) were independent predictors of 28-day mortality in the indirect ARDS group. Creatinine, PT, and OI have interactions with ARDS types (all *p* < 0.01). *Conclusions*: The patients with direct ARDS were more likely to be older and male and have worse conditions and prognoses than those with indirect ARDS. Creatinine, PT, and OI were predictors of 28-day mortality only in the indirect ARDS group. The differences between direct and indirect ARDS suggest the need for different management strategies of ARDS.

## 1. Introduction

Acute Respiratory Distress Syndrome (ARDS) is characterized by severe hypoxemia due to noncardiogenic pulmonary edema [1]. Despite advances in our understanding of ARDS and its treatment strategies, the mortality of patients with ARDS remains unacceptably high [2,3]. Many of studies have made attempts to determine the predictors of mortality in ARDS, with conflicting results [3,4,5,6]. For instance, there are different results regarding whether diabetes mellitus (DM) affects the clinical outcomes of ARDS. It was found that the mortality was lower in an experiment exploring the relationship of DM and ARDS [7] and higher in a predictive model of lung injury [8], and there was no correlation between DM and ARDS in a prognosis-related study [9]. One possible explanation for these discrepant results is the clinical heterogeneity of ARDS [10].

This high heterogeneity has been recognized as a barrier to the effective treatment of patients with ARDS [11,12]. To reduce the high heterogeneity, there have been growing efforts to categorize ARDS into subtypes (e.g., direct and indirect ARDS) according to various characteristics. The classification of direct and indirect ARDS is based on the risk factors of ARDS [11,13,14]. Pneumonia, aspiration, and lung contusion are attributed to direct ARDS, and pancreatitis, non-pulmonary sepsis, trauma, and burns are attributed to indirect ARDS [11]. Hoelz et al. showed that direct ARDS causes more alveolar collapse, fiber exudation, and alveolar wall edema than indirect ARDS [15]. Calfee et al. suggested that there are more severe alveolar epithelial damages and less endothelial damage in direct ARDS than in indirect ARDS [16]. 

Whether there are differences in clinical characteristics and mortality between direct and indirect ARDS is still controversial [17,18], and so it needs further research and discussion. Therefore, we aimed to explore the different clinical characteristics and predictors of 28-day mortality between direct and indirect ARDS.

## 2. Materials and Methods

### 2.1. Study design

This study was approved by the Ethical Committee of the Second Affiliated Hospital of Chongqing Medical University (No.2022-36). Because of the retrospective nature of this study, the requirement for informed consent was waived by the ethics committee. To ensure confidentiality, all patient information was anonymously recorded.

**Figure 1:**
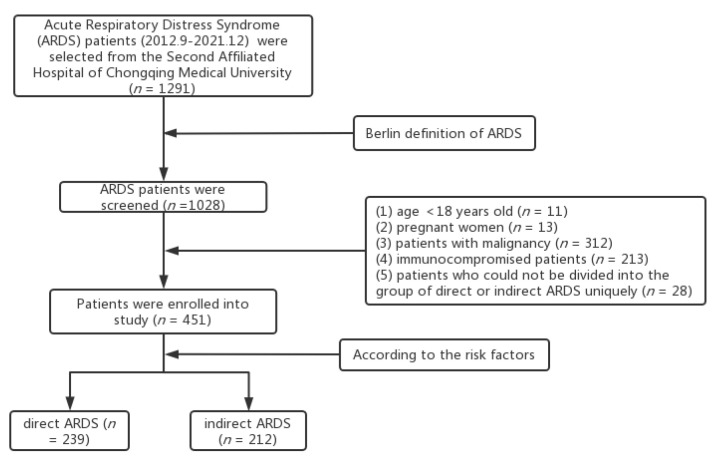
Flow chart.

In this study, 1291 ARDS patients were enrolled between September 2012 and December 2021 at the Second Affiliated Hospital of Chongqing Medical University, according to the International Classification of Diseases (ICD) code. We further screened all patients according to the Berlin criteria [19]. Patients who had lost important data with being related to the Berlin criteria could not be enrolled in the study. All patients who met the following criteria were excluded: (1) age of < 18 y, (2) pregnant women, (3) patients with malignancy, (4) immunocompromised patients, and (5) patients who could not be divided into the group of direct or indirect ARDS uniquely. Finally, 451 patients were enrolled in the study (Figure 1). According to the different risk factors, each patient was divided into the direct ARDS group (*n* = 239; pneumonia (*n* = 227), aspiration of gastric contents (*n* = 3), and lung contusion (*n* = 9)) or the indirect ARDS group (*n* = 212; pancreatitis (*n* = 136), non-pulmonary sepsis (*n* = 36), trauma (*n* = 35), burns (*n* = 2), and poisoning (*n* = 3)). In this study, the primary outcome was 28-day mortality. Survivors were defined as patients who were alive 28 days after admission. 

### 2.2. Data Collection

We reviewed the electronic medical records of all study subjects and collected demographic information, clinical characteristics, comorbidity, laboratory findings, significant scores, and therapy used. All laboratory findings were collected from medical records within 24 h of ARDS diagnosis. If laboratory findings were recorded multiple times in the first 24 h after the diagnosis of ARDS, we used the laboratory findings from the first measurement. According to the medical records, patients discharged within 28 days were followed-up by telephone, and those with lost contact information were not enrolled in the study.

### 2.3. Statistical Analysis

SPSS software (version 26.0, IBM, United States) and R statistical software (version 3.4.2, R Core Team, New Zealand) were used to perform the statistical analysis and plotting. Continuous variables and categorical variables were represented by medians (interquartile ranges) and frequencies (percentages), respectively. Continuous variables were employed the Mann–Whitney U test to compare groups, conversely, using the chi-square test. Regression imputation was used to fill in the missing values of some laboratory findings. 

Multivariate logistic regression analysis was applied to determine the predictors of 28-day mortality. Variables that were statistically significant (*p* < 0.05) between the survivors and non-survivors in the two ARDS groups were enrolled into the multivariate logistic regression analysis. Scores (APACHE II and SOFA) were excluded from the multivariate logistic regression analysis because of the overlap of demographic information, laboratory findings, and scores. Because the age-adjusted Charlson Comorbidity Index (aCCI) overlaps with age and comorbidity, it was not included in multiple logistic regression analysis. To further identify the different predictors of 28-day mortality between the two ARDS groups, we used the interaction terms between the ARDS type and risk factors. Adjusted odds ratios (OR) with 95% CI were reported, and we used a two-tailed *p*-value of < 0.05 to define statistical significance.

## 3. Results

### 3.1. Baseline Characteristics of Direct and Indirect ARDS

In the direct ARDS group, patients were more likely to be older (*p* < 0.001) and male (*p* = 0.009) and have a lower oxygenation index (OI, *p* < 0.001), higher aCCI (*p* < 0.001), and more comorbidity (including hypertension, chronic obstructive pulmonary disease (COPD), and coronary artery disease, all *p* < 0.05) compared with the indirect ARDS group (Table 1). In the indirect ARDS group, the values of C-reactive protein (CRP, *p* < 0.001), procalcitonin (PCT, *p* < 0.001), blood glucose (*p* < 0.001), and prothrombin time (PT, *p* = 0.003) were higher than those of the direct ARDS group. There were higher acute physiology and chronic health evaluation (APACHE) II scores (*p* = 0.001), higher 28-day mortality rates (*p* < 0.001), and shorter lengths of stay (*p* < 0.001) in the direct ARDS group than in the indirect ARDS group. In terms of therapy, patients with direct ARDS used more glucocorticoids, more vasopressors, and less rehabilitation (all *p* < 0.05). More details are shown in Table 1.

As patients with moderate ARDS accounted for the majority, we conducted a subgroup analysis that limited the research scope to moderate ARDS (oxygenation index of 100–200 mmHg). The results revealed that the difference in baseline information between moderate direct and indirect ARDS was similar to that of the original group (Supplementary Table S1).

### 3.2. Clinical Characteristics of Survivors and Non-Survivors

Table 2 shows that, in the direct ARDS group, non-survivors were older (*p* < 0.001), and likely to incorporate MODS (*p* = 0.004) and had higher values of aCCI (*p* = 0.001), creatinine (*p* = 0.006), PT (*p* = 0.002), APACHEII (*p* = 0.003), and sequential organ failure assessment (SOFA, *p* < 0.001). In the indirect ARDS group, there were statistically significant differences in OI, comorbidity, laboratory findings (including platelet, C-reactive protein, procalcitonin, albumin, PT, and activated partial thromboplastin time (APTT)), and important scores of APACHEII and SOFA between the survivors and non-survivors. In both the direct and indirect ARDS groups, there were differences in the use of vasopressors and invasive mechanical ventilation between the survivors and non-survivors. 

### 3.3. Independent Predictors of 28-Day Mortality of Direct and Indirect ARDS

**Figure 2:**
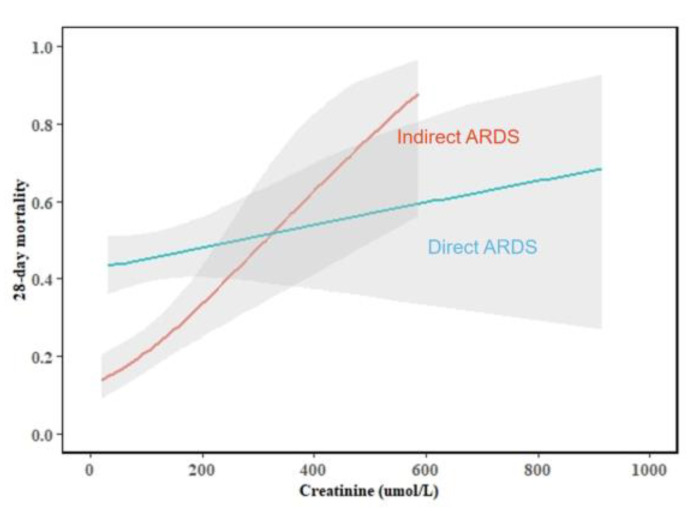
The interaction between creatinine and ARDS type (test for interaction, *p* = 0.001).

**Figure 3:**
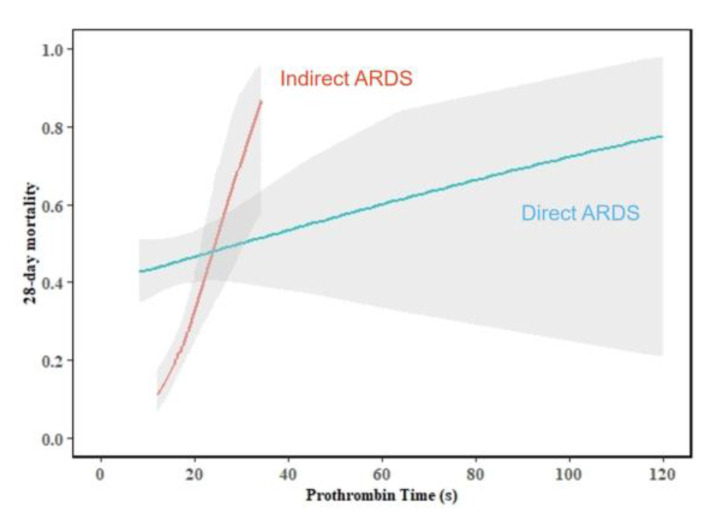
The interaction between prothrombin time and ARDS type (test for interaction, *p* < 0.001).

**Figure 4:**
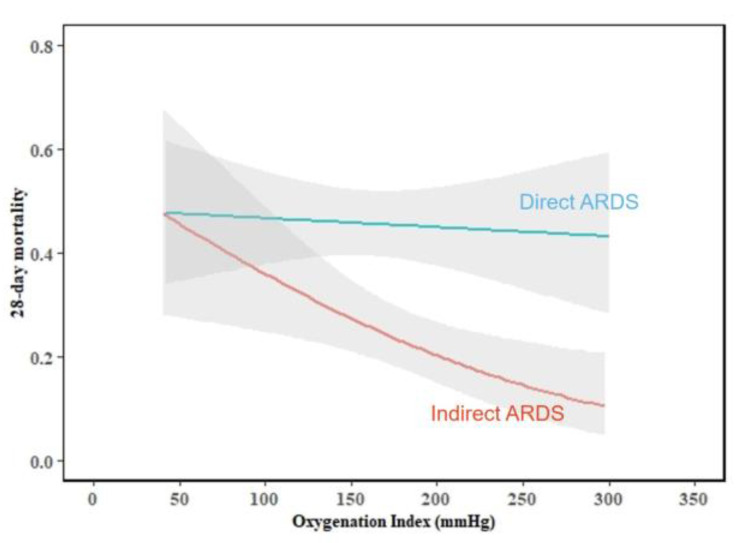
The interaction between oxygenation index and ARDS type (test for interaction, *p* < 0.001).

To determine whether there were different predictors of 28-day mortality in the direct and indirect ARDS groups, we performed multivariate logistic regression analysis and assessed the interactions between the risk factors and the ARDS type (Table 3). In the total group, age, MODS, creatinine, and OI were independently associated with 28-day mortality. In the direct ARDS group, age (OR = 1.032, *p* = 0.002) and MODS (OR = 2.059, *p* = 0.026) were predictors of 28-day mortality. In the indirect ARDS group, age (OR = 1.034, *p* = 0.005), MODS (OR = 4.370, *p* = 0.002), creatinine (OR = 1.005, *p* = 0.017), PT (OR = 1.161, *p* = 0.006), and OI (OR = 0.991, *p* = 0.010) were independent predictors of 28-day mortality. Creatinine (*p* = 0.001), PT (*p* < 0.001), and OI (*p* < 0.001) had interactions with the ARDS type (direct and indirect ARDS) (Figure 2, Figure 3 and Figure 4).

## 4. Discussion

In our study, there were significant differences in the clinical characteristics and independent predictors of 28-day mortality in the direct and indirect ARDS groups. In the direct ARDS group, patients were more likely to be older and male and have a lower oxygenation index and more comorbidity compared with the indirect ARDS group. Patients with direct ARDS used more glucocorticoids and more vasopressors and had less rehabilitation. Patients diagnosed with direct ARDS had higher mortality rates, which has been validated in previous studies [14]. In addition, age and MODS were predictors of mortality in the direct and indirect ARDS groups. PT, creatinine, and OI were independent predictors of 28-day mortality only in the indirect ARDS group. 

In our study, we found that age was an independent predictor of 28-day mortality in the direct and indirect ARDS groups. Other clinical studies have found that ARDS patients of different ages had different outcomes [18,20]. Therefore, our findings are consistent with those of previous studies. Some animal models have suggested that the severity of lung injury varies with age because pulmonary edema and lung tissue damage were aggravated with age [21,22,23]. For example, Manish Bodas found that age-related proteostasis-imbalance can enhance nuclear factor kappa-B mediated inflammatory responses and play a crucial role in acute lung injury [24], suggesting that patients with ARDS can be managed by age stratification. 

Our study found that MODS was an independent predictor of 28-day mortality in the direct and indirect ARDS groups. A previous study confirmed that MODS is a well-recognized major determinant of death in patients with ARDS [25]. In addition, in an animal experiment, Martin found that inhibiting the activity of the intracellular enzyme phosphoinositide-3 kinase gamma can prevent the development of ARDS and MODS and improve survival [26], suggesting that ARDS patients with MODS have a poor prognosis.

In our study, creatinine was an independent predictor of 28-day mortality in the indirect ARDS group. Previous studies have found similar results in that ARDS non-survivors had higher creatinine values than survivors [27,28]. Creatinine is a laboratory indicator that can reflect the severity of renal impairment. Some studies have found that ARDS combined with renal impairment can increase the mortality of ARDS patients because of inflammatory factors and immune disorders [29,30,31]. It is well known that the kidneys are closely linked to the lungs, and both organs are supported by a thick network of capillaries [29]. Previous animal models have found that renal injury can reduce the clearance of inflammatory factors, leading to increased pulmonary neutrophils aggregation and pulmonary capillary permeability, which causes or exacerbates lung injury [29,32,33]. Therefore, our study found that creatinine is only associated with mortality in the indirect ARDS (more endothelial damage) group because renal impairment may be associated with increased pulmonary capillary permeability.

In our study, PT was an independent predictor of 28-day mortality in the indirect ARDS group. Similarly, in a COVID-19-related ARDS study, Denis showed that prolonged PT was a prognostic indicator and patients with prolonged PT were sicker [34]. It is well known that the coagulation system plays an important role in ARDS. Studies have shown that the interrelationship between inflammatory mediators and the coagulation cascade is relevant to the pathogenesis of ARDS [35,36], which may explain the association between PT and ARDS. The reason why PT was a predictor of 28-day mortality only in indirect ARDS group requires further in-depth research. 

The oxygenation index is the main objective indicator of the diagnostic criteria of ARDS, and it is also an indicator for evaluating the severity of ARDS. With in-depth studies of ARDS, some scholars have gradually proposed that the oxygenation index could be affected by other factors when predicting the prognosis of ARDS. For example, Sunitha Palanidurai et al. found that the multifactorial P/FP ratio has a greater predictive validity for hospital mortality in ARDS than does the oxygenation index [37]. Similarly, our study showed that OI is only associated with the death of indirect ARDS patients, but not with direct ARDS patients, suggesting that the prognosis of ARDS predicted by oxygenation index may be different due to different causes of ARDS, but the mechanism is still unclear, More large-scale studies are required to confirm our findings.

Interestingly, we found that the CRP of non-survivors was lower than that of survivors. In our study, all laboratory findings were collected from medical records within 24 h of ARDS diagnosis, which is more reflective of the early stage of diseases. Consistent with our results, Bajwa E.K. et al. found that the increasing plasma levels of CRP within 48 h of ARDS onset were associated with improved survival, lower organ failure scores, and fewer days of mechanical ventilation [38], and some possible reasons could account for this, for example, biologically, neutrophils play a crucial role in lung injury. Buchta R found that CRP inhibited chemotaxis, along with other characteristic neutrophil functions [39]. Mechanistically, the CRP-mediated inhibition of p38 mitogen-associated protein kinase activity reduced the neutrophil signal transduction proteins involved in the response to chemotactic stimuli [40]. In an animal experiment, Heuertz R.M. et al. found that by reducing neutrophil influx and protein leakage, CRP played a protective role in neutrophil-mediated lung injury [41]. Taken together, CRP may have a protective effect against ARDS.

In our study, we confirmed that several common variables (including age and MODS) are risk factors for mortality in ARDS patients. To our knowledge, this is one of the few studies to explore the significant differences in the clinical characteristics and 28-day mortality between direct and direct ARDS patients. Specially, laboratory findings were enrolled into multivariate logistic regression analysis to investigate the predictors of 28-day mortality. 

In addition, our study has limitations. Firstly, this study carries the inherent limitations of any retrospective study, such as missing data and selection bias, and we have tried to expand the sample size to reduce the bias. Secondly, patients with moderate ARDS accounted for the majority and there was proportional inequity of survivors to non-survivors in our study, and so we conducted a subgroup analysis and obtained similar results. Thirdly, because of the retrospective nature of this study, we were unable to establish causality between variables and increased mortality in ARDS. Fourthly, our study was a single-center study, and our demographic data were geographically restricted. These factors may limit the generalizability of our findings. Therefore, large prospective studies with more complete clinical data and in vitro/in vivo studies for mechanisms are needed to validate our findings.

## 5. Conclusions

In the direct ARDS group, patients were more likely to be older and male and have worse conditions and prognoses than those in the indirect ARDS group. By multivariate logistic regression analysis, it was found that age and MODS were predictors of 28-day mortality in the direct and indirect ARDS groups, whereas creatinine, PT, and OI were predictors of 28-day mortality only in the indirect ARDS group. The differences between direct and indirect ARDS suggest the need for different management strategies of ARDS.

## Figures and Tables

**Table 1:** Baseline characteristics of direct and indirect ARDS.

Characteristic	Total ARDS (*n* = 451)	Direct ARDS (*n* = 239)	Indirect ARDS (*n* = 212)	*p-*Value
Male, *n* (%)	300 (66.5)	172 (72.0)	128 (60.4)	0.009
Age (years)	63.0 (47.0–75.0)	70.0 (59.0–78.0)	50.0 (41.3–66.0)	< 0.001
Alcohol, *n* (%)	132 (29.3)	64 (26.8)	68 (32.1)	0.217
Oxygenation index (mmHg)	171.0 (127.5–215.0)	154.1 (118.2–203.0)	184.5 (141.8–227.8)	< 0.001
**Comorbidity, *n* (%)**				
Diabetes, *n* (%)	101 (22.4)	60 (25.1)	41 (19.3)	0.143
Hypertension, *n* (%)	146 (32.4)	101 (42.3)	45 (21.2)	< 0.001
Coronary heart disease, *n* (%)	57 (12.6)	48 (20.1)	9 (4.2)	< 0.001
COPD, *n* (%)	50 (11.1)	47 (19.7)	3 (1.4)	< 0.001
MODS, *n* (%)	107 (23.7)	62 (25.9)	45 (21.2)	0.240
aCCI	4.0 (2.0–5.0)	5.0 (3.0–6.0)	2.0 (1.0–4.0)	< 0.001
**Laboratory findings**				
Platelet (×109/L)	165.0 (109.0–228.0)	173.0 (109.0–252.0)	158.5 (108.3–215.0)	0.115
NLR	13.0 (7.7–21.7)	12.5 (6.8–21.0)	14.0 (8.4–22.2)	0.235
CRP (ng/mL)	135.6 (55.4–200.0)	102.2 (34.7–176.3)	182.4 (97.6–200.0)	< 0.001
Procalcitonin (ng/mL)	1.5 (0.4–8.4)	0.9 (0.2–6.5)	2.6 (0.6–12.7)	< 0.001
Albumin (g/L)	30.2 (25.8–34.0)	30.2 (25.1–33.6)	30.4 (26.8–34.4)	0.113
Blood glucose (mmol/L)	8.7 (6.6–12.3)	8.1 (6.3–11.0)	9.8 (7.4–14.0)	<0.001
Creatinine (umol/L)	76.1 (52.4–121.6)	79.1 (56.6–120.8)	73.7 (50.3–122.2)	0.147
PT (s)	15.1 (14.0–17.0)	14.8 (13.8–16.4)	15.5 (14.1–17.4)	0.003
APTT (s)	42.1 (36.9–47.7)	41.7 (36.5–47.2)	42.2 (37.2–48.1)	0.221
**Scores**				
SOFA	6.0 (4.0–8.0)	6.0 (4.0–8.0)	6.0 (4.0–9.0)	0.360
APACHEⅡ	18.0 (14.0–23.0)	19.0 (15.0–24.0)	16.0 (12.0–21.0)	0.001
**Therapy, n (%)**				
IMV, *n* (%)	152 (33.7)	81 (33.9)	71 (33.5)	0.928
Glucocorticoid, *n* (%)	181 (45.1)	147 (61.5)	34 (16.0)	< 0.001
Vasopressor, *n* (%)	185 (41.0)	116 (48.5)	69 (32.5)	0.001
Rehabilitation, *n* (%)	298 (66.1)	140 (58.6)	158 (74.5)	< 0.001
**Outcome**				
28-day mortality, *n* (%)	158 (35.0)	109 (45.6)	49 (23.1)	< 0.001
Hospital LOS, *n* (d)	15 (7–24)	14 (6–22)	18 (9–28)	< 0.001

Note: Continuous variables were displayed by using medians (interquartile ranges) and compared by using the Mann–Whitney U test. Categorical variables were displayed by using frequencies (percentages) and compared by using the chi-square test. Abbreviations: TOA = time of ARDS diagnosis after admission. COPD = chronic obstructive pulmonary disease. MODS = multiple organ dysfunction syndrome. CRP = C-reactive protein. NLR = neutrophil-to-lymphocyte ratio. PT = prothrombin time. APTT = activated partial thromboplastin time. SOFA = sequential organ failure assessment. APACHE = acute physiology and chronic health evaluation. LOS = length of stay. IMV = invasive mechanical ventilation. aCCI = age-adjusted Charlson Comorbidity Index.

**Table 2:** Clinical characteristics of the survivors and non-survivors.

Characteristic	Direct ARDS (*n* = 239)		*p*-Value	Indirect ARDS (*n* = 212)		*p-*Value
	Survivors (*n* = 130, 54.4%)	Non-Survivors (*n* = 109, 45.6%)		Survivors (*n* = 163, 76.9%)	Non-Survivors (*n* = 49, 23.1%)	
Male, *n* (%)	88 (67.7)	84 (77.1)	0.108	97 (59.5)	31 (63.3)	0.637
Age (years)	66.0 (56.0–75.0)	74.0 (64.5–82.0)	< 0.001	50.0 (41.0–63.0)	52.0 (41.5–78.5)	0.096
Alcohol, *n* (%)	31 (23.8)	33 (30.3)	0.264	55 (33.7)	13 (26.5)	0.343
Oxygenation index (mmHg)	160.8 (121.4–195.3)	143.9 (111.0–210.3)	0.593	188.0 (152.0–230.0)	174.0 (117.0–213.0)	0.024
**Comorbidity, *n* (%)**						
Diabetes, *n* (%)	35 (26.9)	25 (22.9)	0.479	35 (21.5)	6 (12.2)	0.152
Hypertension, *n* (%)	59 (45.4)	42 (38.5)	0.285	35 (21.5)	10 (20.4)	0.873
Coronary heart disease, *n* (%)	22 (16.9)	26 (23.9)	0.183	5 (3.1)	4 (8.2)	0.121
COPD, *n* (%)	27 (20.8)	20 (18.3)	0.639	1 (0.6)	2 (4.1)	0.072
MODS, *n* (%)	24 (18.5)	38 (34.9)	0.004	21 (12.9)	24 (49.0)	< 0.001
aCCI	4.0 (3.0–5.0)	5.0 (4.0–6.0)	0.001	2.0 (1.0–4.0)	3.0 (1.0–5.0)	0.014
**Laboratory findings**						
Platelet (×109/L)	175.0 (123.0–248.8)	167.0 (98.0–256.5)	0.579	164.0 (115.0–216.0)	133.0 (70.5–208.5)	0.027
NLR	12.8 (7.6–21.1)	12.0 (6.1–22.0)	0.333	13.7 (8.1–21.1)	15.2 (8.8–28.2)	0.214
CRP (ng/Ml)	95.7 (34.4–179.0)	116.5 (37.0–172.9)	0.706	200.0 (116.4–200.0)	148.7 (53.8–200.0)	0.006
Procalcitonin (ng/mL)	0.7 (0.2–6.4)	1.4 (0.2–7.0)	0.575	1.8 (0.5–7.1)	6.9 (1.1–20.4)	0.007
Albumin(g/L)	30.6 (26.1–34.1)	28.6 (24.3–33.5)	0.102	30.5 (27.9–35.0)	28.6 (24.3–32.9)	0.015
Blood glucose (mmol/L)	8.1 (6.4–11.0)	8.2 (6.0–11.3)	0.697	9.7 (7.4–13.8)	10.1 (7.8–14.6)	0.355
Creatinine (umol/L)	70.1 (55.2–100.8)	91.1 (60.6–136.9)	0.006	64.0 (47.8–98.5)	127.4 (74.6–224.3)	< 0.001
PT (s)	14.6 (13.5–15.8)	15.3 (14.1–17.5)	0.002	15.3 (14.1–16.6)	17.6 (14.9–21.1)	< 0.001
APTT (s)	41.7 (36.1–46.9)	42.1 (37.0–48.3)	0.508	41.4 (36.7–47.1)	46.2 (37.5–67.7)	0.005
**Scores**						
SOFA	5.0 (4.0–7.0)	7.0 (4.0–9.0)	< 0.001	5.0 (4.0,8.0)	10.0 (6.5,12.0)	< 0.001
APACHEⅡ	17.5 (14.0–22.0)	21.0 (16.0–24.5)	0.003	15.0 (11.0,19.0)	25.0 (17.5,30.5)	< 0.001
**Therapy, *n* (%)**						
IMV, *n* (%)	35 (26.9)	46 (42.2)	0.013	37 (22.7)	34 (69.4)	< 0.001
Glucocorticoid, *n* (%)	73 (56.2)	74 (67.9)	0.063	24 (14.7)	10 (20.4)	0.342
Vasopressor, *n* (%)	33 (25.4)	83 (76.1)	< 0.001	29 (17.8)	40 (81.6)	< 0.001
Rehabilitation, *n* (%)	79 (60.8)	61 (56.0)	0.453	121 (74.2)	37 (75.5)	0.857

Note: Continuous variables were displayed by using medians (interquartile ranges) and compared by using the Mann–Whitney U test. Categorical variables were displayed by using frequencies (percentages) and compared by using the chi-square test. See Table 1 legend for expansion of abbreviations.

**Table 3:** Independent predictors of 28-day mortality of direct and indirect ARDS.

Characteristic	Total ARDS (*n* = 451)		Direct ARDS (*n* = 239)		Indirect ARDS (*n* = 212)		*p*-Value *
	Adjusted OR (95% CI)	*p-*Value	Adjusted OR (95% CI)	*p-*Value	Adjusted OR (95% CI)	*p*-Value	
Age (years)	1.034 (1.021–1.048)	< 0.001	1.032 (1.012–1.053)	0.002	1.034 (1.010–1.059)	0.005	
MODS	2.972 (1.802–4.902)	< 0.001	2.059 (1.092–3.879)	0.026	4.370 (1.755–10.882)	0.002	
Platelet (×109/L)	1.000 (0.998–1.002)	0.949	1.000 (0.998–1.002)	0.838	1.000 (0.996–1.004)	0.999	
CRP (ng/mL)	0.997 (0.994–1.000)	0.054	0.999 (0.996–1.003)	0.791	0.996 (0.991–1.002)	0.193	
Procalcitonin (ng/mL)	1.000 (0.997–1.004)	0.843	1.003 (0.996–1.010)	0.396	0.995 (0.986–1.004)	0.235	
Albumin(g/L)	0.970 (0.936–1.005)	0.089	0.981 (0.938–1.027)	0.413	0.976 (0.916–1.040)	0.456	
Creatinine (umol/L)	1.002 (1.000–1.004)	0.049	1.001 (0.998–1.003)	0.637	1.005 (1.001–1.010)	0.017	0.001
PT (s)	1.022 (0.992–1.053)	0.148	1.010 (0.983–1.038)	0.460	1.161 (1.043–1.292)	0.006	< 0.001
APTT (s)	1.003 (0.994–1.011)	0.510	1.005 (0.990–1.020)	0.555	0.994 (0.980–1.008)	0.388	
Oxygenation index (mmHg)	0.996 (0.992–0.999)	0.023	0.999 (0.995–1.004)	0.810	0.991 (0.984–0.998)	0.010	< 0.001

Note: Multivariate logistic regression was performed in the total ARDS cohort and then separately in the direct ARDS and indirect ARDS populations. The interactions of the ARDS type (direct or indirect) with creatinine, PT, and OI were included in the regression analysis. * *p*-value for interaction with ARDS type.

## Data Availability

The datasets used in the current study are available from the corresponding author upon reasonable request.

## Supplementary Materials

The following supporting information can be downloaded at: https://www.mdpi.com/article/10.3390/medicina58111563/s1, Table S1: Baseline Characteristics of the Moderate Direct and Indirect ARDS.
