# Supplementary material for: Comparison of Clinical Characteristics and Predictors of Mortality between Direct and Indirect ARDS
Source: Medicina (Kaunas). 2022 Oct 30;58(11):1563. doi: 10.3390/medicina58111563 (PMC9697068; doi:10.3390/medicina58111563)
Supplement: Supplementary file 1 [file medicina-58-01563-s001.zip › medicina-1908545-supplementary.pdf]

Table S1. Baseline Characteristics of the Moderate Direct and Indirect ARDS.

Characteristic	Total ARDS (n = 253)	Direct ARDS (n = 141)	Indirect ARDS （n = 112）	p Value
Male, n (%)	157 (62.1)	99 (70.2)	58 (51.8)	0.003
Age (years)	64.0 (50.0-75.0)	68.0 (59.0-77.0)	53.0 (44.3-66.8)	<0.001
Alcohol, n (%)	68 (26.9)	35 (24.8)	33 (29.5)	0.408
Oxygenation index (mmHg)	151.4 (127.8-176.5)	145.5 (124.8-171.6)	164.0 (132.3-180.0)	0.011
Diabetes, n (%)	54 (21.3)	36 (25.5)	18 (16.1)	0.068
Hypertension, n (%)	87 (34.4)	61 (43.3)	26 (23.2)	0.001
Coronary heart disease, n (%)	33 (13.0)	28 (19.9)	5 (4.5)	<0.001
COPD, n (%)	31 (12.3)	29 (20.6)	2 (1.8)	<0.001
MODS, n (%)	51 (20.2)	34 (24.1)	17 (15.2)	0.078
aCCI	3.0 (2.0-5.0)	4.0 (3.0-6.0)	2.0 (1.0-4.0)	<0.001
Platelet (×109 /L)	164.0 (106.0-220.0)	275.0 (108.5-248.0)	158.0 (99.3-204.3)	0.095
NLR	13.5 (8.5-23.8)	12.1 (6.9-23.6)	14.5 (9.3-25.7)	0.115
CRP (ng/mL)	137.6 (70.4-200.0)	108.5 (53.8-175.8)	200.0 (113.2-200.0)	<0.001
Procalcitonin (ng /mL)	1.5 (0.3-8.2)	1.0 (0.2-7.0)	2.4 (0.7-9.9)	0.006
Albumin (g/L)	29.4 (25.3-33.3)	29.5 (25.7-32.9)	39.4 (25.7-32.9)	0.952
Blood glucose (mmol/L)	8.5 (6.6-11.4)	8.0 (6.4-10.2)	9.5 (6.8-13.7)	0.002
Creatinine (umol/L）	74.6 (51.6-108.3)	79.1 (56.1-109.8)	68.0 (48.2-104.6)	0.073
PT (s)	15.0 (14.0-16.9)	14.7 (13.7-16.6)	15.5 (14.1-17.2)	0.019
APTT (s)	42.4 (37.3-47.2)	41.6 (36.8-47.3)	42.2 (37.4-47.3)	0.313
SOFA	6.0 (4.0-8.0)	6.0 (3.5-8.0)	6.0 (4.0-9.0)	0.134
APACHE Ⅱ	18.0 (14.0-22.4)	19.0 (15.0-24.0)	17.0 (14.0-21.0)	0.014
IMV, n (%)	81 (32.0)	46 (32.6)	35 (31.3)	0.816
Glucocorticoid, n (%)	107 (42.3)	86 (61.0)	21 (18.8)	<0.001
Vasopressor, n (%)	102 (40.3)	67 (47.5)	35 (31.3)	0.009
Rehabilitation, n (%)	185 (73.1)	95 (67.4)	90 (80.4)	0.021
28-Mortality, n (%)	77 (30.4)	54 (38.3)	23 (20.5)	0.002

Hospital LOS, *n* (d)	16 (8-24)	15 (7-22)	20 (11-29)	0.001
